# Fluorescent Light Energy in the Management of Multi Drug Resistant Canine Pyoderma: A Prospective Exploratory Study

**DOI:** 10.3390/pathogens11101197

**Published:** 2022-10-18

**Authors:** Andrea Marchegiani, Alessandro Fruganti, Marilena Bazzano, Matteo Cerquetella, Fabrizio Dini, Andrea Spaterna

**Affiliations:** School of Biosciences and Veterinary Medicine, University of Camerino, 62024 Matelica, Italy

**Keywords:** dog, deep pyoderma, interdigital furunculosis, multi-resistant infection, fluorescent light energy

## Abstract

The increase in prevalence of staphylococcal antimicrobial resistance has been also associated with pyoderma in dogs, and prolonged antibiotic treatment, as often needed in severe cases of pyoderma, has been related to influencing possible development of multidrug resistance (MDR). Fluorescent light energy (FLE) has been indicated to improve pyoderma lesions as adjunct therapy to systemic antibiotics. In the present study, we evaluated the effect of FLE on clinical signs of MDR canine deep pyoderma (CDP) and interdigital furunculosis (CIF) when administered as solely management. Sixteen client-owned dogs affected by CIF (five dogs) and CDP (eleven dogs) were scored using a dedicated scoring system and received a single FLE applications twice weekly, until clinical resolution was achieved. Mean time to achieve complete resolution was 5.20 ± 3.56 weeks (median 3 weeks) for CIF cases and 4.18 ± 1.47 weeks (median 4 weeks) for CDP ones. FLE shows promise as an aid to managing clinical signs while reducing reliance on antibiotics for MDR CDP and CIF. In this study, FLE was responsible for the decrease in lesion scores and resolution of MDR pyoderma infection without any adjunct therapy, having a potential useful role to play in antibiotic stewardship programs, efficiently promoting complete clinical resolution of MDR lesions while optimizing the use of antibiotics.

## 1. Introduction

Canine pyoderma is a family of cutaneous disorders frequently diagnosed in small animal practice [1,2], conventionally treated with both topical and systemic antibiotics [3,4]. Managing canine pyoderma is becoming more difficult due to the presence of multidrug-resistant (MDR) staphylococci which are progressively encountered by veterinary practitioners, especially in dermatologic area [5,6] In the past few years, veterinary staff are facing the burden of increased antimicrobial resistance [5] Multi Resistant *Staphylococcus Pseudintermedius* (MRSP) has been diagnosed mainly in pyoderma and otitis in dogs, cats, and horses whose prevalence varies according to the geographical location [7,8] It has been estimated a MRSP prevalence varying from 15.6 to 17% in USA up to 67% in dogs presenting pyoderma in Japan [9,10,11] In dogs, MRSP infection has been found to have an estimated prevalence of 7% [12] which is expected to raise, augmenting the risk of MDR transmission in domestic environment [13] Multidrug resistance initiated and incited a remarkable change in philosophy of systemic therapy and especially oral antibiotic treatment [14,15] MDR has also enhanced the necessity for new medications intended for local use and with antibiotic-sparing effect for MDR strains [16] Local care for bacterial skin diseases has been an innovation of veterinary dermatology since its establishment, but for a long time, it has been supposed to be only an add-on to oral antibiotics, regarded as key therapy [17].

In the context of topical therapies, photobiomodulation (PBM) is a non-pharmacological procedure explored in canine dermatology in which different wavelengths of visible light, produced by a dedicated device (chromophore gel and LED lamp) are administered to interfere with cellular activity to enhance healing [18]. Fluorescent light energy (FLE) is a pioneering form of PBM that uses fluorescence to decrease the expression of tumor necrosis factor alpha (TNF alpha) and increase epidermal growth factor (EGF), fibroblast growth factors (FGFs), transforming growth factor beta (TGF beta), platelet-delivered growth factor (PDGF), vascular endothelial growth factor (VEGF), collagen I and III, Ki67, factor VIII, and decorin (DCN) levels, in addition to causing an increase in both number and size of mitochondria [19,20]. FLE has been demonstrated to ameliorate and cure superficial bacterial folliculitis [18], deep pyoderma [21], canine interdigital furunculosis (CIF) [22], canine perianal fistulas [23], and cutaneous calcinosis [24]. In such instances, FLE was responsible for a lessening in the length of antibiotic therapy (and time needed for healing) when administered as add-on therapy, especially in deep and interdigital pyoderma.

In the present study, we investigated the results of the FLE on clinical manifestations of multidrug resistant deep pyoderma and interdigital furunculosis in dogs when administered as solely management.

## 2. Materials and Methods

The dogs enrolled in the study were all client-owned pets conducted to the Veterinary Teaching Hospital (VTH) of University of Camerino for investigation of pyoderma. The study protocol was compliant with European legislation on the protection of animals used for scientific purposes and approved by the University of Camerino Body for Protection of Animals (Prot. N. 1/2017); in addition, dogs’ owners fulfilled and signed an informed consent form before participation in the study. No experimental animals were used in this study. There was no restriction of age, breed, bodyweight, or sex in animals recruited, but pregnant dogs were not enrolled in this study.

To be pre-enrolled, history and clinical signs had to be coherent with a presumptive diagnosis of deep pyoderma (CIF or CDP)—exhibiting a combination of typical clinical lesions including papules even crusted, haemorrhagic vesicles and crusts, as well as ulcers, erosions, and draining fistulae. At least one of the clinical lesions assessed based on previous published charts (global lesion score, GLS) [21,22] had to be scored 3 or 4 (severe or very severe) on a 0 (normal skin)–4 (highest grade of severity) scale. Bacteriological samples were obtained from fistulae, ulcers or erosions and from the underside crusts, when present; to be enrolled, a multi-resistant bacterium had to be isolated on subsequent culture and susceptibility testing. Ectoparasites and *Malassezia* spp. infection were ruled out by skin scraping, impression smears, and tape stripping cytology while dermatophytosis were discarded based on fungal culture. In addition, a neutrophils engulfing score (NES) on a 0–4 scale (0 = none seen; 1 = less than 1 neutrophils engulfing bacteria; 2 = between 1 and 4 neutrophils engulfing bacteria; 3 = between 5 and 10 neutrophils engulfing bacteria; 4 = more than 11 neutrophils engulfing bacteria per high powered field, ×500 magnification over 10 fields) was applied. 

Complete blood count and blood chemistries were performed to identify dogs with signs of systemic ill health or leishmaniasis; endocrine serology tests were performed to rule out thyroid, adrenal gland and endocrine pancreas disorders. Flea products were allowed during the study while systemic antibiotics, antihistamines, corticosteroids, ciclosporin, as well as topical FANS or antimicrobials had to be discarded at least the two weeks before enrolment. Lokivetmab and oclacitinib were inaccessible during the study period. 

FLE (Phovia®, Vetoquinol France) system comprised the illumination for 2 min of a photoconverter gel layer (approx. 2 mm in thickness, put on the lesions present) with the LED lamp (non-coherent blue light, 440–460 nm peak wavelength, 55–129 mW/cm^2^ power density) holding the lamp at an approximated distance of 5 cm. Once illuminated, the gel was removed and FLE was administered twice weekly, three to four days apart, until complete clinical resolution, intended as complete disappearance of lesions initially present (GLS scores had been reduced to 0, absent) was achieved.

Clinical assessments (GLS and NES) were accomplished at enrolment (day 0) and then weekly for ten weeks. When a dog reached clinical resolution, a further 4-month observational period begun, to assess for possible recurrence of lesions in the same previously affected site. 

All data analysis (Student’s *t*-test, Fisher test, Mann–Whitney tests) were run using SAS v9.4 and values of *p* ≤ 0.05 were considered significant. The weekly reduction in GLS and NES scores were analyzed using non-parametric methods based on change from baseline and Mann–Whitney tests. 

## 3. Results

All dogs enrolled completed the study and no withdrawals were recorded. Mixed breeds were overrepresented for both CDP and CIF, while German Shepherd dogs were the most represented purebred (Table 1). Long-coated dogs were more frequent than short-coated. 

FLE did not cause any adverse events in neither group. All dogs improved their condition and achieved complete clinical healing in an average time of 4.18 ± 1.47 and 5.20 ± 3.56 weeks for CDP and CIF, respectively.

Regarding the results of microbiological swabs performed on lesions, the predominant MDR pathogens isolated were *S. pseudintermedius* (*n* = 8) and *S. aureus* (*n* = 8). *Streptococcus* spp. (*n* = 6), *Enterococcus* (*n* = 3), *Bacillus* (*n* = 1), and *Proteus* (*n* = 1) were occasionally detected. 

**Figure 1:**
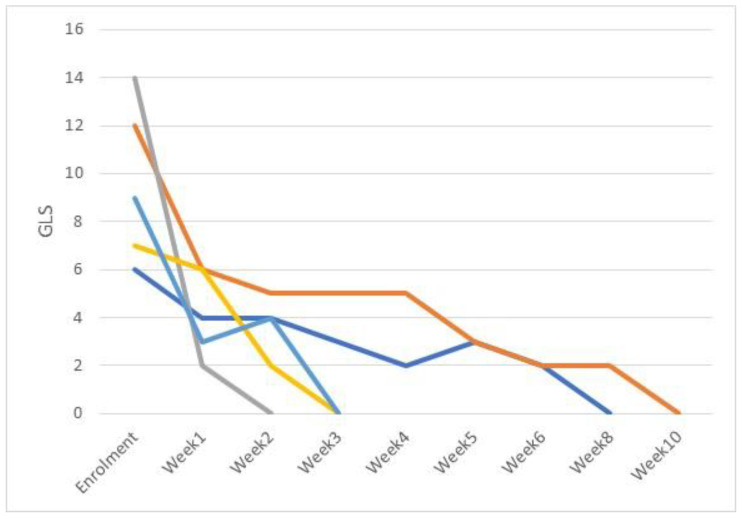
Weekly fluctuation of Global Lesion Scores (GLS), CIF cases (5 dogs).

**Figure 2:**
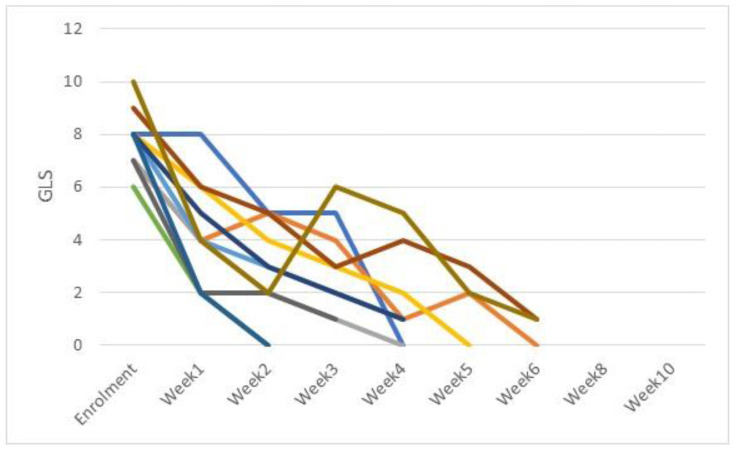
Weekly fluctuation of Global Lesion Scores (GLS), CDP cases (11 dogs).

As a secondary endpoint, the variation in clinical severity of CIF and CDP lesions and NES per week are shown in Figure 1 and Figure 2 for CIF and CDP, respectively. 

GLS decreased significantly in CIF cases from week 1 and maintained this trend for the duration of the healing process, except for week 4 where there was a slight increase in GLS, (Figure 1). For CDP cases, a statistically significant decrease in GLS was seen from week 1 and maintained until clinical resolution was achieved (Figure 2). At week 6, average global lesion score was 0.67 for CDP dogs and 2.0 for those affected by CIF. These results are superimposable to those of previous published studies in which FLE was applied as adjunct treatment to systemic antibiotic [21,22].

**Figure 3:**
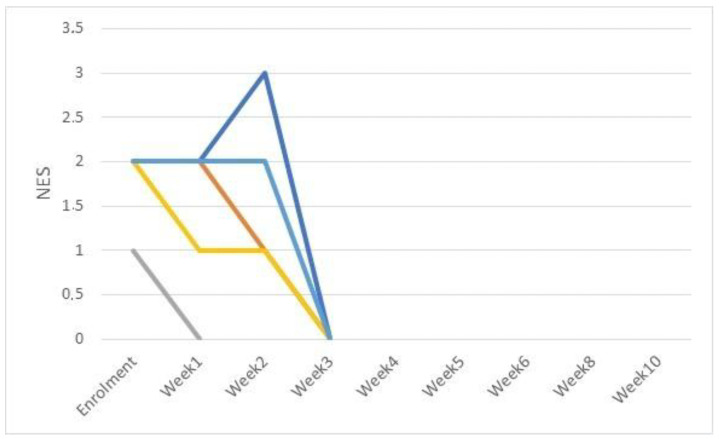
Neutrophil Engulfing Bacteria (NES) scores for CIF cases (5 dogs).

**Figure 4:**
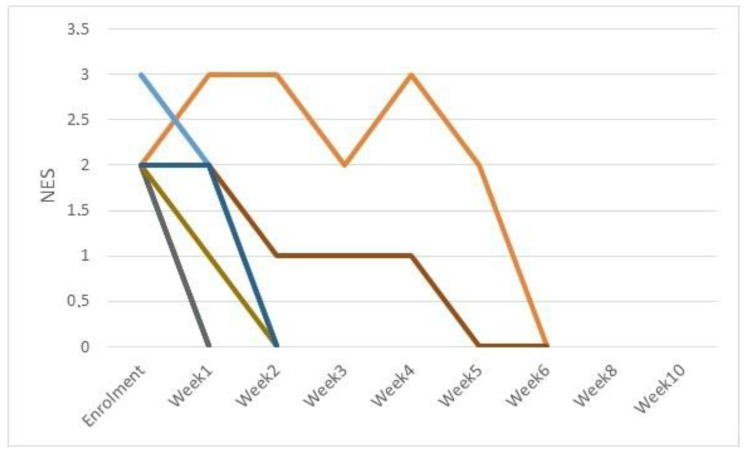
Neutrophil Engulfing Bacteria (NES) scores for CDP cases (11 dogs).

NES scores (ranging from 0 to 4) are shown in Figure 3 and Figure 4 for CIF and CDP, respectively. A statistically significant decrease in average NES score (*p* < 0.0001) was seen in week 3 and 1 for CIF and CDP dogs, respectively.

All dogs were enrolled for follow-up period and no recurrence of both CIF and CDP was recorded in the 4-month assessment period. 

## 4. Discussion

CDP and CIF are common and potentially debilitating diseases of dogs, usually secondary to other medical conditions which, if not properly addressed and managed, tend to become perpetuating in nature and likely to compromise the efficacy of the antimicrobial protocol [3,5]. Long-term successful management of CDP and CIF are essential to prevent recurrence: despite that clinical manifestation may improve after few weeks of antimicrobial therapy [25], lengthy antibiotic administration (8 to 12 weeks) is frequently necessary in cases of severe CIF and it has been related to influencing possible development of MDR [26,27,28,29]. 

Antibiotic treatments of inadequate duration or with empirically chosen molecules may predispose to the development of MDR and facilitates the transmission of these bacterial strains from pets to humans [30].

Bacterial culture and sensitivity testing is frequently ignored in CDP and CIF diagnostic workup for which empiric therapy is still erroneously considered suitable in certain cases, particularly during first-time or untreated infections [5] Some authors have demonstrated that selected antimicrobials widely considered to be effective during deep bacterial skin infection (i.e., β-Lactam, macrolides, lincosamides, and potentiated sulfonamides) may promote establishment of methicillin-resistant strains [7,31]. On the other hand, other authors highlighted a possible efficacy of rifampicin for the treatment of multidrug resistant staphylococcal infections, requiring concurrent topical antimicrobial administration to obtain a complete resolution in near 80% of dogs, despite more studies to assess the safety profile are needed [32]. Many MDR staphylococcal isolates of the present study resulted to be resistant to fluoroquinolones as previously demonstrated by other authors during MDR infections [7]. In addition, fluoroquinolone exposure has been identified as a potential cause for MRSA isolation in dogs and humans, through different mechanisms including augmented exposure to colonization by fluoroquinolones-resistant bacteria and bond to host cells [33,34,35].

Antimicrobial options for the treatment of MDR pyoderma are often limited and topical therapy could be the sole treatment for CIF and CDP [17,31]. It is also now well accepted by the international scientific community that long-term antibiotic use (more than 5 weeks) carries a high-risk factor for the development of antibiotic resistant bacteria; thus, treatment protocols that reduce the systemic antibiotic treatment duration are desirable [26,36].

The increasing identification of MDR skin infections has led to a vivid revolution in thinking of veterinary antibiotic treatment, heightening a need for a different strategies and devices in local antibacterial treatment [17]. An increasing number of chlorhexidine, hypochlorous, benzoyl, and different topical antibacterial, not antibiotic, products are available for pyoderma management in small animal practice as topical formulation [17].

Antibacterial activity of blue light has been already tested using an in-vitro setting, to ascertain its ability to control bacterial growth of also multi-resistant bacterial, but with scarce to mild results [37,38,39,40]. Blue light is used by FLE to produce fluorescence and studies have shown that the modulation of inflammatory mediators and stimulation of growth factors release are only observed in response to the combination of gel and lamp illumination; conversely, the blue LED lamp alone has no effect [19]. In previous published studies, FLE controlled chronic inflammatory condition (as CDP and CIF tend to become) significantly improving the clinical outcome as add-on to oral antibiotics, being able to lessen the time needed for healing and accordingly the time of oral antibiotic treatment to less than half [21,22]. 

The lack of a control group (managed with other topical product) and the low number of enrolled dogs represent limitations of the present study. Despite this, the results obtained suggest that FLE may be considered as an option for the management of CDP and CIF. Since the use of topical products would be likely to be one of the future approaches in the management of bacterial skin disease, further studies enrolling more patients and eventually with control group are needed. 

## 5. Conclusions

FLE can be considered as the sole therapeutic option in case of multidrug resistant CDP and CIF, promoting complete clinical resolution of lesions. It has the benefits of being an antibiotic-sparing agent in MDR infections, promoting public and animal health, and avoiding the spreading of antibiotic resistance. 

## Figures and Tables

**Table 1:** Description of signalment data for enrolled dogs.

	CDP	CIF
**Breed** (*n*)		
mixed breed	5	2
German Shepherd	4	1
English Setter	1	
Italian Bracco		1
Labrador		1
Pittbull	1	
**Coat**		
long *(n)*	7	4
short *(n)*	4	1
**Weight**		
mean (kg)	29.00	35.00
sd (kg)	9.47	8.54
**Body condition score** (BCS)		
mean	4.55	5.60
sd	0.82	0.89

## Data Availability

Data in the present study are covered by confidentiality.
